# 15‐Year Follow‐Up of Multidisciplinary Management of Severe External Root Resorption Caused by Biocortically Impacted Maxillary Canine: A Case Report

**DOI:** 10.1155/crid/5169817

**Published:** 2026-02-26

**Authors:** Lara Maalouf, Philip Farha, Elie Amm

**Affiliations:** ^1^ Department of General Dentistry, Boston University Henry M. Goldman School of Dental Medicine, Boston, USA, bu.edu; ^2^ Private Practice, Beirut, Lebanon; ^3^ Private Practice, Boston, USA; ^4^ Department of Orthodontics, Boston University Henry M. Goldman School of Dental Medicine, Boston, USA, bu.edu

**Keywords:** canine impaction, case report, endodontic microsurgery, severe external root resorption

## Abstract

**Aim:**

To highlight the effectiveness of a conservative, multidisciplinary approach in managing external root resorption caused by a biocortically impacted maxillary canine in a young patient and to demonstrate the potential for long‐term preservation of a compromised central incisor.

**Summary:**

A 14‐year‐old female presented with a biocortically impacted maxillary right canine causing a severe root resorption of the adjacent central incisor. Cone‐beam computed tomography (CBCT) revealed direct contact between the impacted canine and incisor root, with only one‐third of the incisor’s root structure intact. Surgical removal of the canine was followed by endodontic microsurgery on the central incisor with minimal apical resection and mineral trioxide aggregate (MTA) for root‐end filling. Internal bleaching addressed MTA‐induced discoloration, and comprehensive orthodontic and esthetic treatments were completed. Over a 15‐year follow‐up, the incisor remained functional, asymptomatic, and stable, demonstrating that early diagnosis and conservative multidisciplinary management can preserve compromised teeth, maintain alveolar bone, and support successful orthodontic treatment.

**Summary**

• Provide insights into the management of severe external root resorption.
• Highlights the critical role of interdisciplinary collaboration in managing complex cases of root resorption.
• Demonstrates the effectiveness of conservative treatment in achieving long‐term preservation of tooth structure, function, and esthetics.

## 1. Introduction

Tooth impaction is defined as the failure of a tooth to erupt into its functional position within the expected developmental period [1]. After third molars and lower premolars, the maxillary canine is the most commonly impacted tooth [2], with a prevalence of 1.7% to 4.7% [3]. Impactions are most associated with anomalies of the adjacent lateral incisors, such as agenesis or peg‐shaped crowns, which can disrupt normal eruption guidance [4].

Impacted canines are usually classified as either buccal or palatal in position [5, 6]. However, ~6.6% are in the mid‐alveolar or bi‐cortical region, where they are neither buccal nor palatal [6]. These biocortically impacted canines, particularly located in sectors 4 or 5 according to Ericson and Kurol’s classification [7], pose an increased risk of inducing severe external root resorption in adjacent incisors due to their proximity and abnormal eruption path [8].

External root resorption is a silent, irreversible process involving progressive loss of cementum and dentin. It frequently affects the middle and apical thirds of the root, is typically asymptomatic, and is detected incidentally on radiographs [9].

Although impacted canines and their sequelae are well documented, reports of bi‐cortical impactions causing extreme resorption of incisors are rare, and there is limited evidence on long‐term outcomes and conservative intervention. This case describes the multidisciplinary management of a 14‐year‐old female with a biocortically impacted canine causing severe resorption of the adjacent central incisor. The report emphasizes the novelty of preserving an incisor with only one‐third of its root length and demonstrates principles that may be generalized to similar high‐risk cases.

The uniqueness of this case lies in the long‐term preservation of a maxillary central incisor that had lost nearly two‐thirds of its root structure due to direct contact with a biocortically impacted canine—a particularly rare impaction pattern. While most impacted maxillary canines are either buccal or palatal, only a small minority are positioned within the mid‐alveolus or bi‐cortical region. Recent cone‐beam computed tomography (CBCT)‐based studies have reported this location in ~3.7%–14.3% of cases, with 8.5% being the most recent documented figure [10, 11]. To our knowledge, this is the first case report describing a 15‐year functional survival of a severely resorbed central incisor managed conservatively following removal of a biocortically impacted canine.

## 2. Case Report

A 14‐year‐old Armenian female presented with the chief complaint of an anterior diastema, a retained maxillary right primary canine (#53), and a peg‐shaped maxillary right lateral incisor (#12). Her medical or dental history was unremarkable, and there were no signs of temporomandibular disorders.

Clinical examination revealed Grade I mobility in teeth #12 and #53, and Grade II mobility in the maxillary right central incisor (#11), according to the Miller Index [12]. All three teeth responded normally to both thermal and electric pulp testing. The patient exhibited a straight lower profile with long‐face syndrome. Intraorally, an Angle Class III molar relationship was noted—3 mm on the right side and 6 mm on the left. The maxillary dental midline aligned with the facial midline, while the mandibular midline deviated 2 mm to the left.

**Figure 1:**
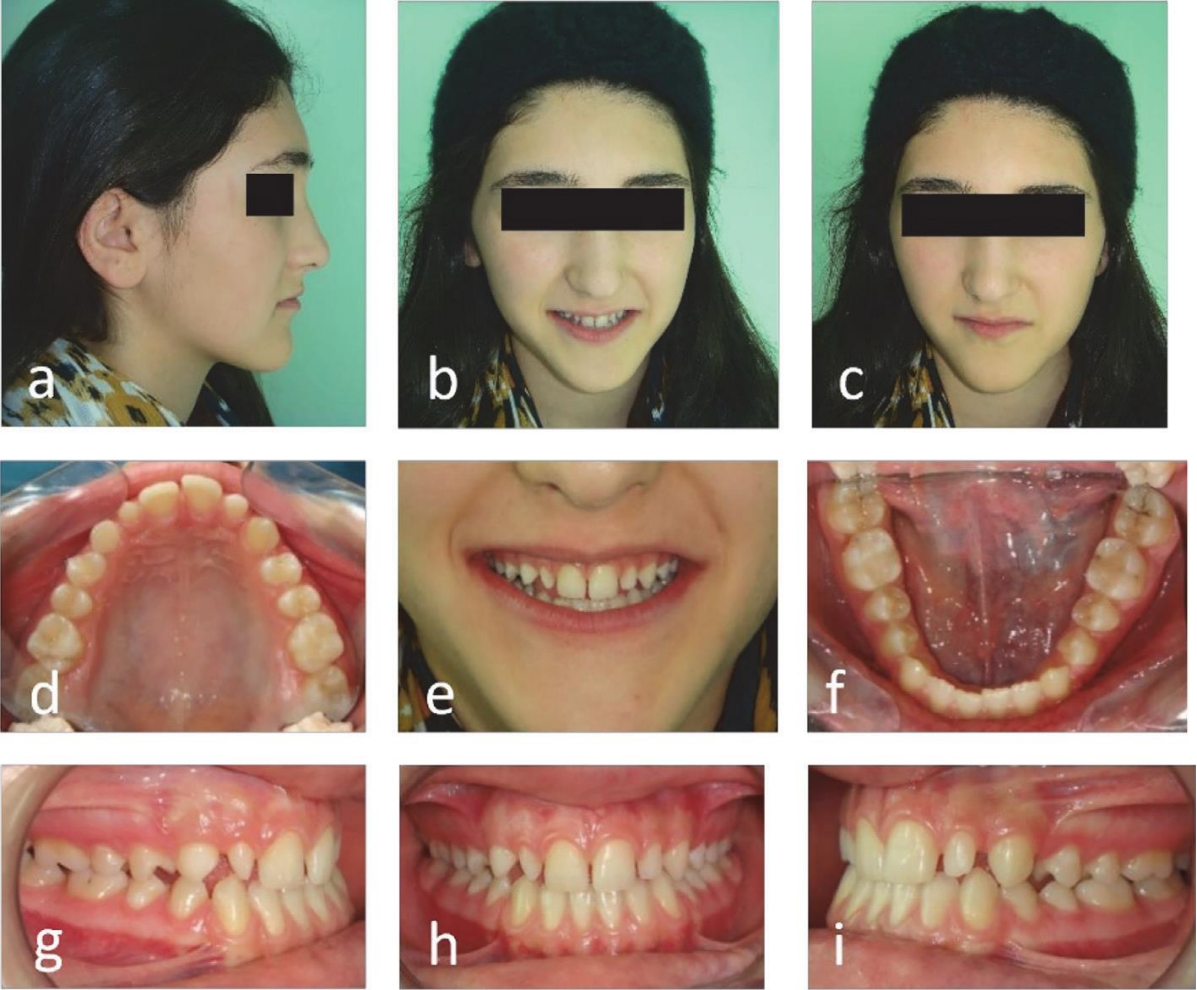
Pretreatment photographs: (a–c) extraoral view with patient’s facial profile and smile; (d–i) intraoral view with anterior diastema, retained primary canine (#53), and peg‐shaped lateral incisor (#12).

**Figure 2:**
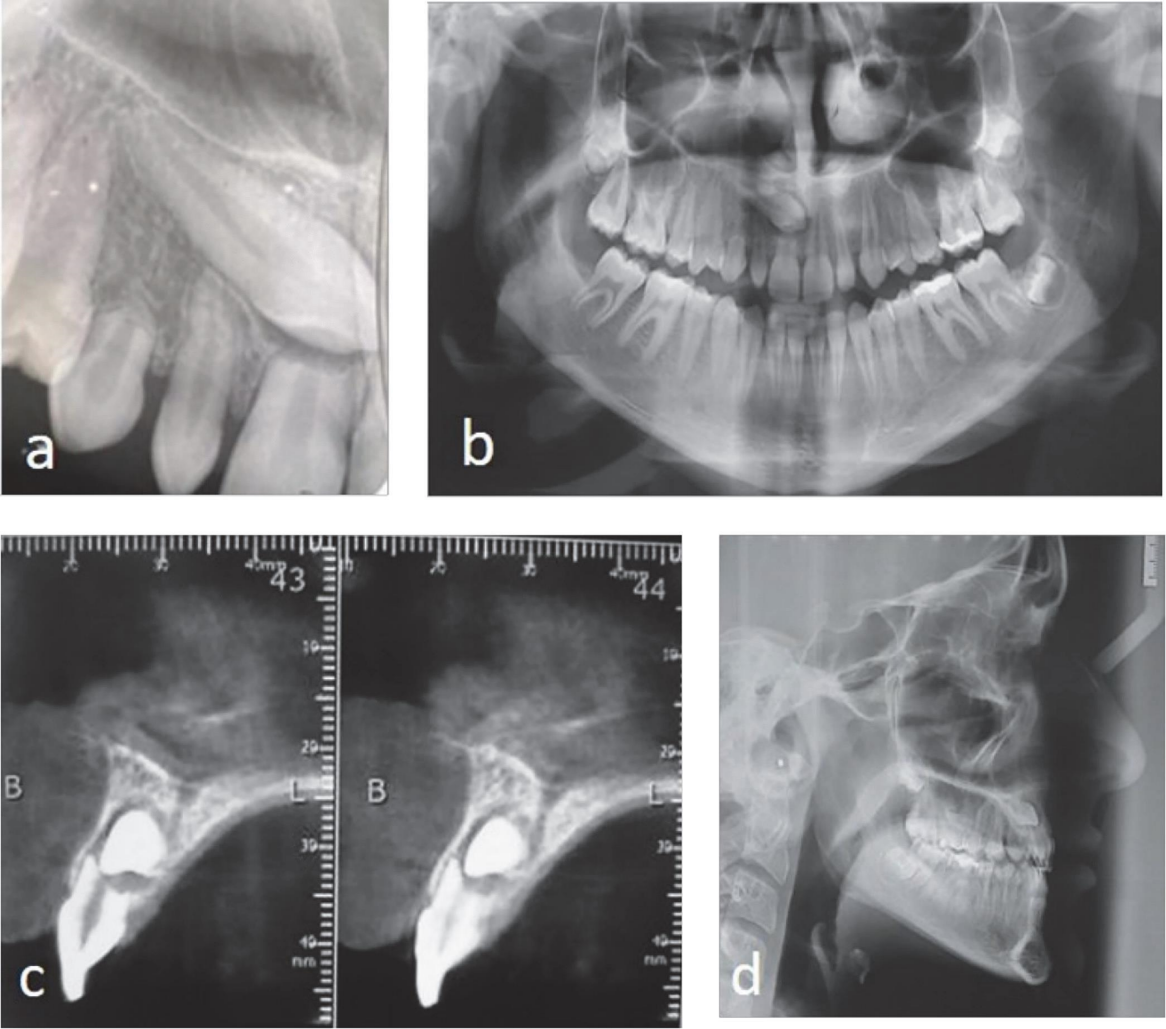
Preoperative radiographic examination: (a) periapical radiograph; (b) panoramic radiograph; (c) cone‐beam computed tomographic scan; and (d) cephalometric radiograph.

Initial clinical photographs (Figure 1a–i) and preoperative radiographs were obtained. The periapical and panoramic radiographs (Figure 2a,b) revealed an impacted maxillary right permanent canine (#13). According to Ericson and Kurol’s classification [7], the impaction was complex: it extended beyond sector 5 horizontally, had an α angle > 45° (sector 3), and was located within the apical third of the adjacent incisor’s root vertically (sector 3).

CBCT (J. Morita, Manufacturing Corp, Kyoto, Japan) with a medium field of view of 80 × 80 mm and a voxel size of 0.125 mm, confirmed a bi‐cortical impaction of the canine, positioned horizontally in the middle of the alveolar process, in direct contact with the right central incisor, leaving only one‐third of the root intact (Figure 2c).

Cephalometric analysis displayed a skeletal Class III pattern with lower incisors contributing to a concave lower profile (Figure 2d).

### 2.1. Treatment Management

Impacted maxillary canines associated with root resorption of adjacent incisors are often diagnosed late due to the absence of symptoms [9]. Although the prognosis of these incisors is guarded, preserving them is preferable in young patients to maintain the alveolar bone ridge and defer complex restorative procedures [13].

The patient and her guardians were presented with treatment options, along with risks and benefits, and informed consent was obtained.

Alternative options discussed included surgical exposure and orthodontic traction of the impacted canine, as well as extraction of the severely resorbed central incisor with future prosthetic/implant replacement. These options were declined due to the high risk of further resorption, periodontal compromise, ankylosis, and the patient’s young age, which contraindicated implant therapy. Preservation of the natural incisor was therefore prioritized.

The primary objective was to preserve the maxillary right central incisor, as loss of a permanent anterior tooth in adolescents poses significant functional, esthetic, and psychological challenges. Conventional restorative options such as implants or fixed partial dentures are contraindicated in growing patients due to ongoing jaw development. Relieving pressure from the impacted canine was necessary to inhibit further progression of external root resorption [14]. Given the risks of surgical exposure and orthodontic traction—including difficulties in alignment, periodontal complications, loss of vitality, further resorption, and ankylosis, particularly in bi‐cortical impactions with canine angulation >31° [7]—surgical extraction of the impacted canine was chosen.

**Figure 3:**
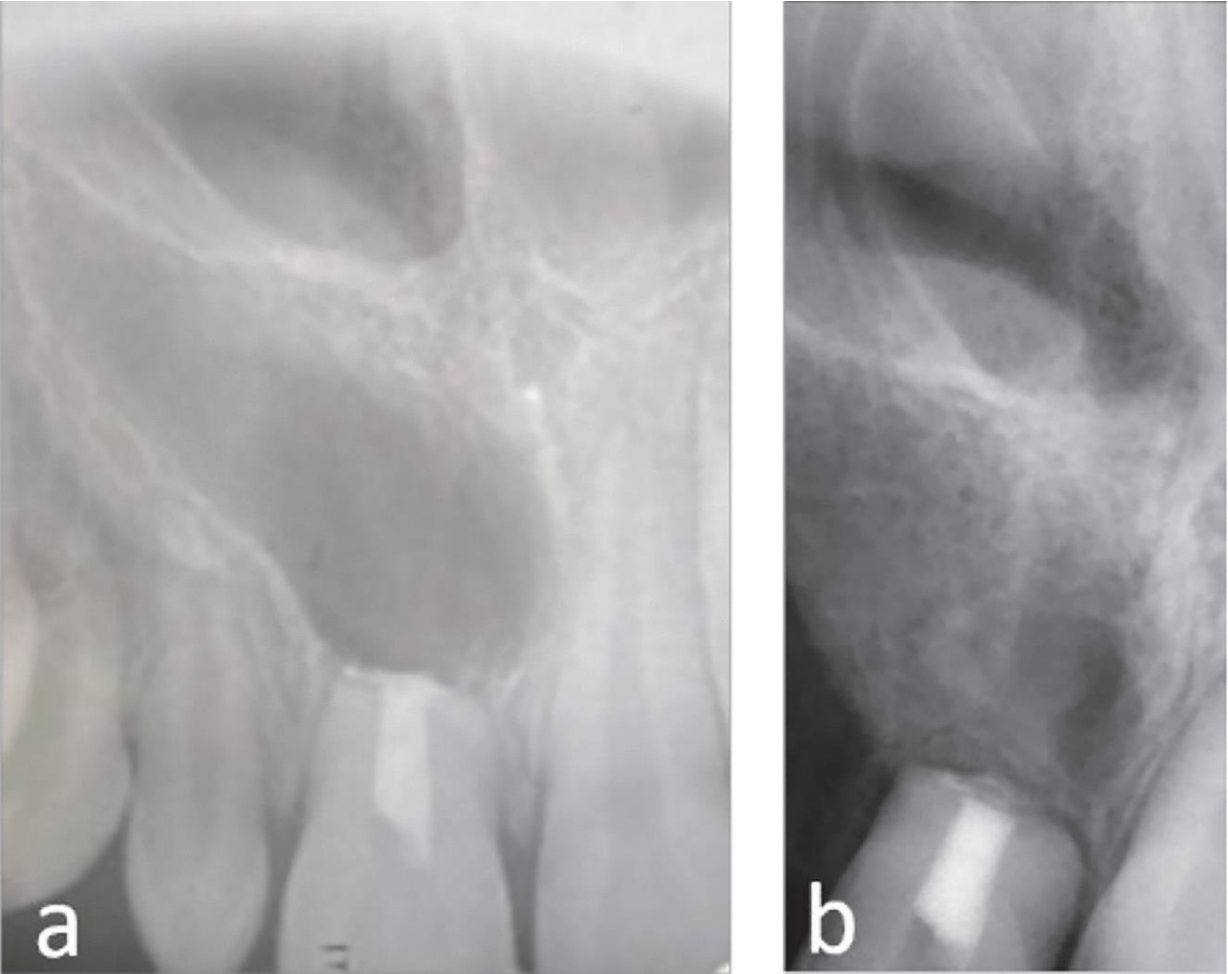
Radiographic documentation of treatment: (a) maxillary right canine extraction and maxillary right central incisor endodontic microsurgery and (b) healing of the maxillary right central incisor.

Endodontic microsurgery was performed under 16× magnification using an operating microscope (Global Surgical, St. Louis, MO). In contrast to the conventional 3‐mm apical resection, the decision was made to perform a minimal resection of less than 1 mm, exceptionally for this case, to preserve the limited remaining root length. The apical surface was refined with an ultrasonic microtip at a 0° bevel, and a retrograde cavity ~2 mm deep was prepared using an ultrasonic retrotip. The cavity was sealed with white mineral trioxide aggregate (MTA Angelus, Brazil) in a single increment and inspected under magnification to verify marginal adaptation [15] (Figure 3a).

**Figure 4:**
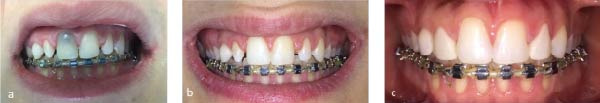
Color management and esthetic restoration: (a) maxillary right central incisor discoloration and lateral incisors prior to composite build‐up; (b) maxillary right central incisor after internal bleaching; and (c) composite build‐up on both lateral incisors.

Extraction of the retained primary canine and future implant placement were also included. Orthodontic treatment commenced after radiographic confirmation of apical healing (Figure 3b). Space closure and arch coordination were completed to optimize conditions for esthetic rehabilitation. Tooth discoloration from apical MTA (Figure 4a) was managed with internal bleaching using sodium perborate powder mixed with saline under a temporary seal. After 10 days, color harmony was restored (Figure 4b). A calcium hydroxide dressing was placed for 1 week to neutralize residual oxygen and alkalize environment, thereby reducing the risk of external cervical resorption [16, 17]. Esthetic restoration of the peg‐shaped maxillary lateral incisors (#12 and #22) was performed using a direct composite layering technique. Teeth were isolated, tissues gently retracted, and bonding surfaces prepared. Multishade composite layering reconstructed enamel and dentin contours, with a slight reduction in mesiodistal (~2 mm) to ensure proportion and symmetry [18]. A final translucent enamel layer, 0.5–1.5 mm coronally relative to the central incisors [19], created a harmonious smile arc (Figure 4c).

### 2.2. Outcomes

**Figure 5:**
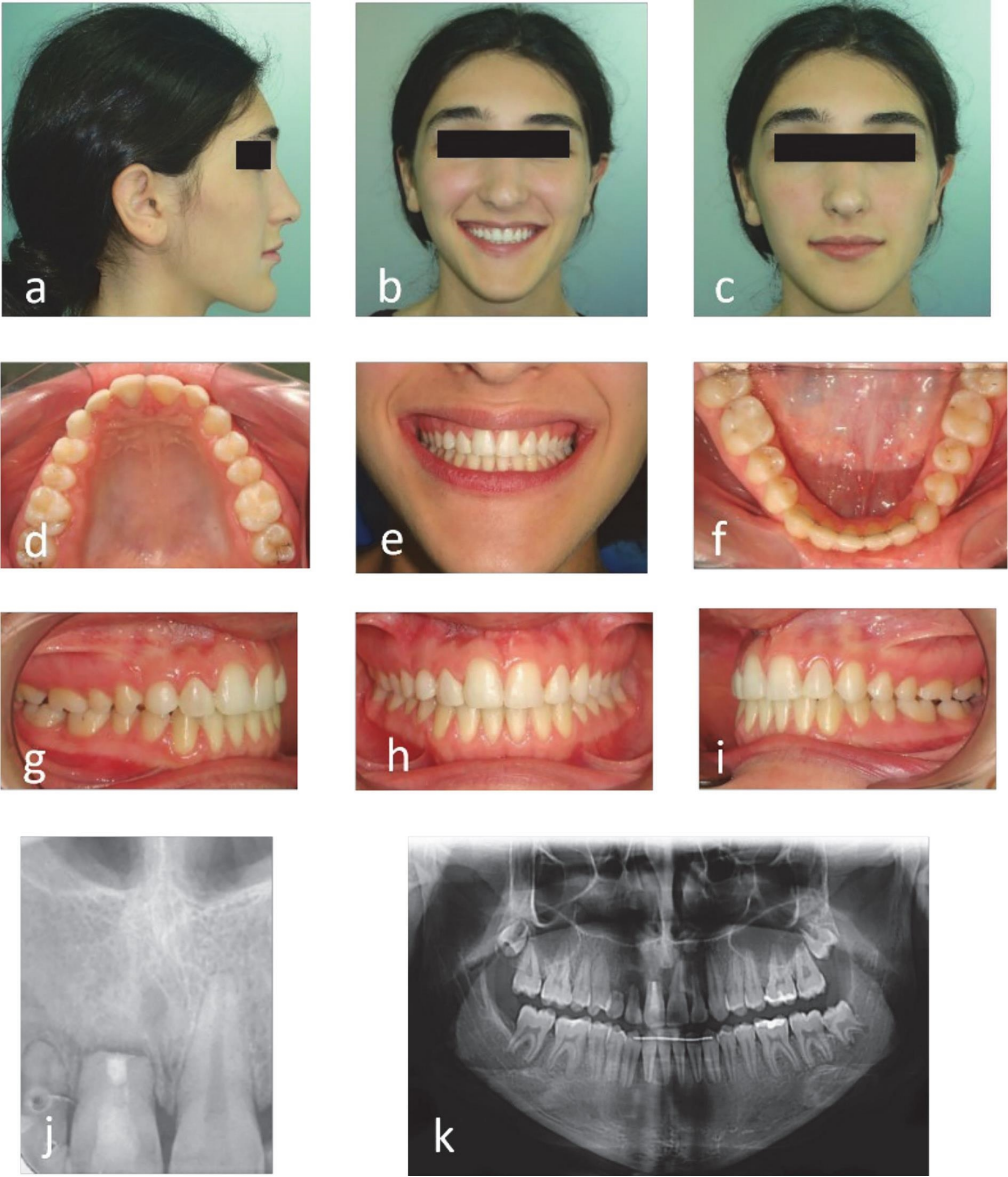
Follow‐up photographs and radiographs 3 years after endodontic microsurgery: (a–c) extraoral view with improved smile esthetics; (d–i) intraoral view with aligned teeth and Class I occlusion; (j) periapical radiograph showing stability of the maxillary right incisor; and (k) panoramic radiograph showing root parallelism and overall dentition.

The patient achieved satisfactory dental and facial esthetics (Figure 5a–c). Dental alignment showed harmonious arch forms, symmetrical arches, and Class I occlusion (Figure 5d–f). The anterior teeth exhibited a positive overjet and overbite of 2 mm each, with acceptable intercuspal contacts (Figure 5g–i). Periapical radiograph confirmed healing at the apex of tooth #11 3 years postmicrosurgery (Figure 5j), and panoramic imaging showed maintained root parallelism (Figure 5k). Some residual root resorption was observed in adjacent teeth, reflecting the severity of the initial pathology and underscoring the importance of early intervention.

Periodontal evaluation throughout follow‐up revealed stable probing depths (≤3 mm), absence of bleeding on probing, stable gingival margins, and no increase in tooth mobility. Clinical attachment levels remained unchanged, supporting long‐term periodontal stability.

Psychosocially, the patient displayed increased confidence and a more positive outlook, attributed to improved dental esthetics and function.

**Figure 6:**
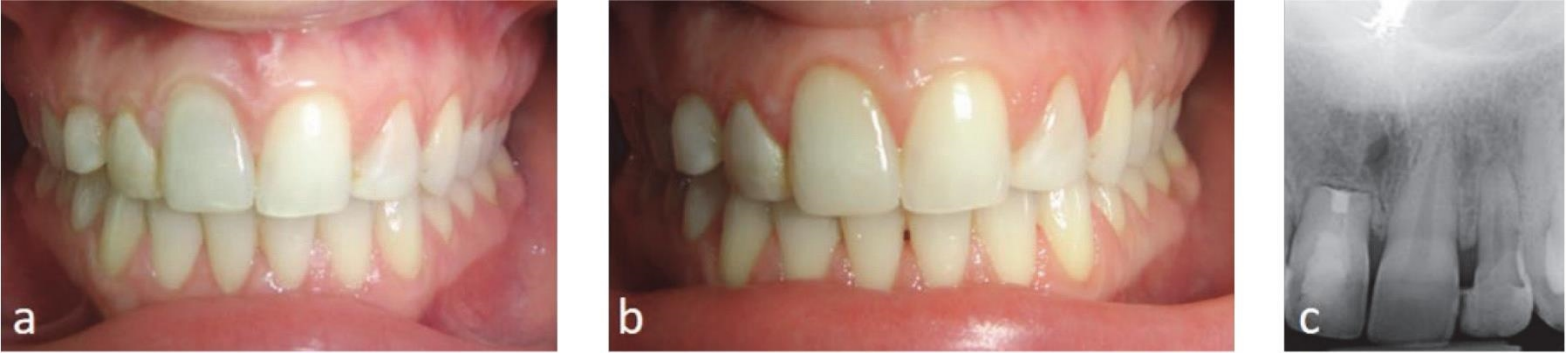
Follow‐up photographs and radiographs 6 years after endodontic microsurgery: (a) mild discoloration 3 years after initial internal bleaching; (b) after repeat internal bleaching; and (c) periapical radiograph showing stable external root resorption.

Mild discoloration of the central incisor recurred 3 years after initial bleaching and 6 years postmicrosurgery. Repeated internal bleaching using the same protocol (Figure 6a,b) was successful, and radiographs demonstrated stable external resorption (Figure 6c).

**Figure 7:**
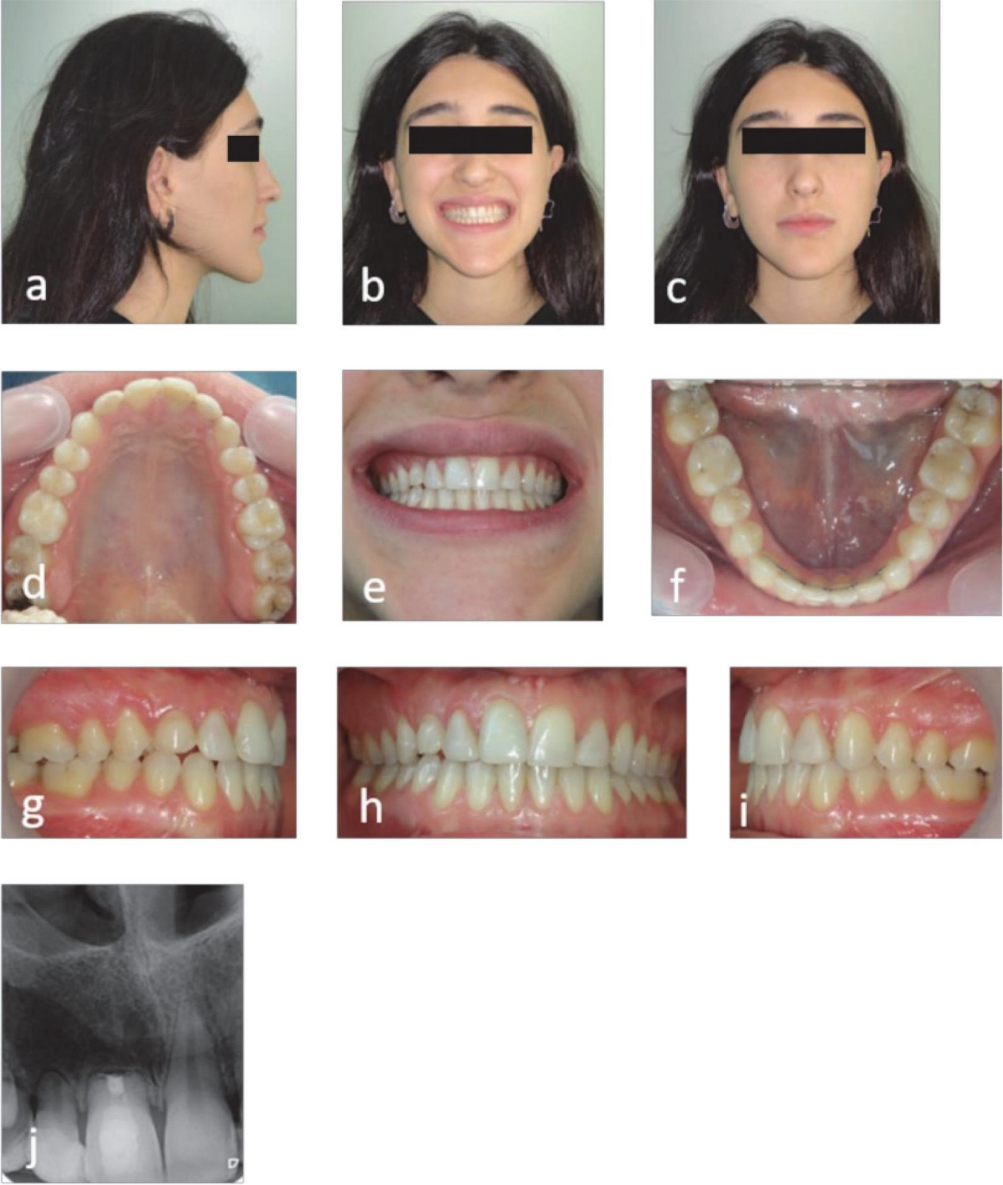
Follow‐up photographs and radiographs 10 years after endodontic microsurgery: (a–c) extraoral view with harmonious facial esthetics and smile; (d–i) intraoral view with aligned arches and Class I occlusion; and (j) periapical radiograph showing stability of the maxillary right incisor.

Clinical and radiographic assessments at 10 (Figure 7a–j) and 15 (Figure 8a–j) years post microsurgery confirmed long‐term stability with maintained esthetic and functional results despite the severe initial resorption. Key treatment milestones and outcomes are summarized in Table 1.

**Figure 8:**
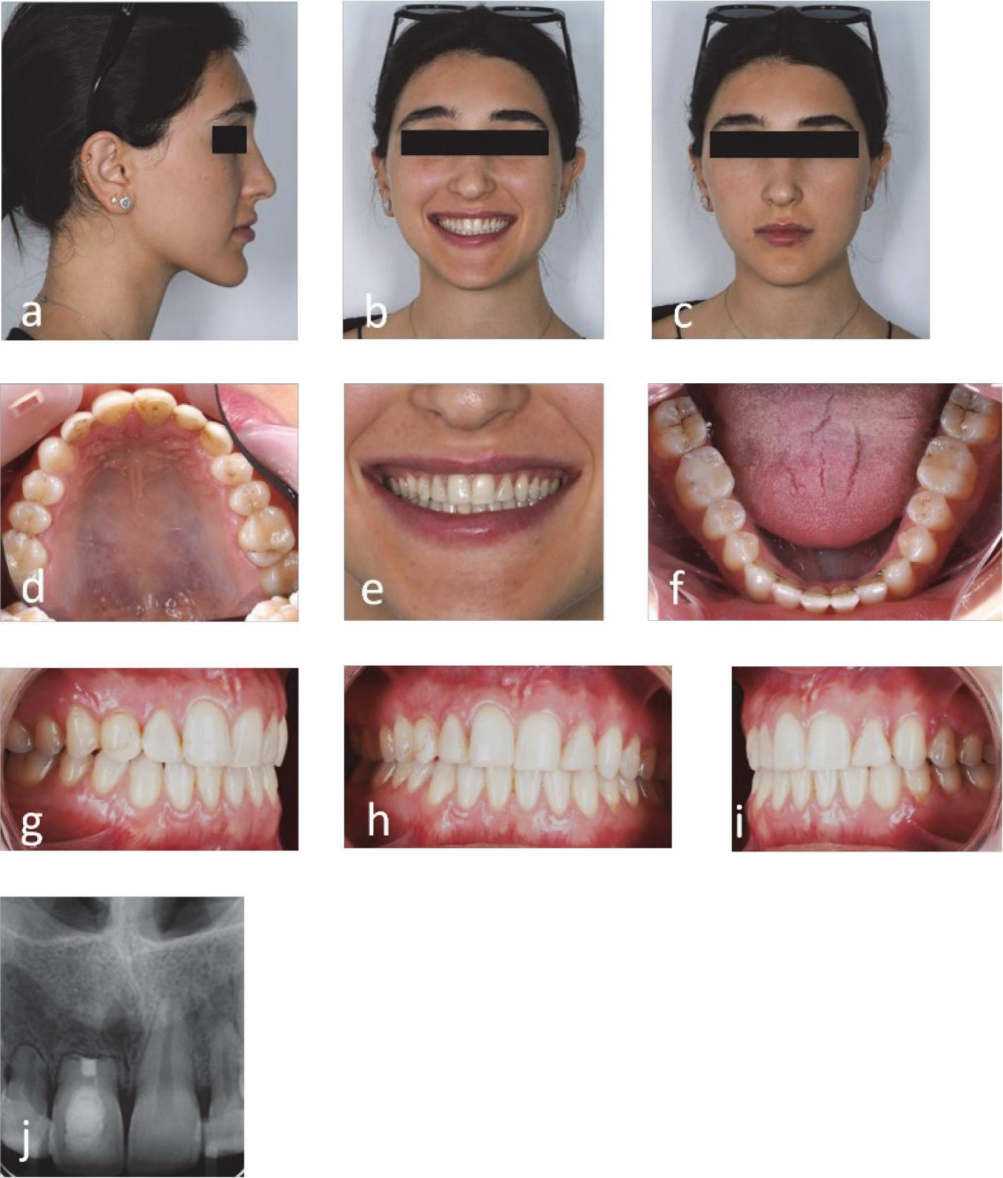
Follow‐up photographs and radiographs 15 years after endodontic microsurgery: (a–c) extraoral view with harmonious facial esthetics and smile; (d–i) intraoral view with aligned arches and Class I occlusion; and (j) periapical radiograph showing stability of the maxillary right incisor.

**Table 1:** Summary of treatment milestones and outcomes over 15 years.

Year	Clinical phase	Key procedures	Radiographic findings	Functional/esthetic outcome
0	Diagnosis	CBCT localization, extraction of impacted canine, and endodontic microsurgery	Severe resorption, one‐third root remaining	Tooth preserved and asymptomatic
0.5	Orthodontic phase	Space closure, arch coordination	Healing of apex confirmed	Stable alignment
3	Esthetic phase	Internal bleaching, composite build‐up	Apical healing	Harmonious smile
6	Follow‐up	Repeat bleaching for mild discoloration	Stable resorption	Maintained function
10	Long‐term review	Routine evaluation	No progression of resorption	Stable occlusion
15	Final evaluation	Clinical and radiographic review	Integrity maintained	Functional and esthetic success

## 3. Discussion

Impacted maxillary canines are frequently associated with complications, including root resorption of adjacent teeth, particularly when palatally displaced [8, 20, 21]. In this case, CBCT revealed a high‐difficulty impaction of the upper right canine (#13), characterized by severe angulation (> 45°), sagittal midline overlap, and close root proximity. According to the Pitt et al. [22] index, these features confer a high difficulty score. A canine–incisor’s root distance ≤ 1 mm is a significant risk factor for resorption [12] supporting the decision to extract the impacted canine rather than attempt orthodontic traction.

The main treatment objective was preservation of the severely resorbed central. Given the limited root length and the patient’s age, endodontic microsurgery with minimal apical resection and MTA was selected. MTA’s bioactive properties, including stimulation of cementogenesis and apical healing, were pivotal to long‐term stabilization [15].

Internal bleaching with sodium perborate mixed with saline successfully managed discoloration linked to MTA migration. This technique is conservative, with lower risk of external cervical resorption compared with peroxide‐based agents [16]. A calcium hydroxide Pulpdent dressing (Pulpdent Corp, Watertown, MA) further optimized bleaching and reduced resorptive risk [17].

CBCT was indispensable for diagnosis, providing 3‐D visualization of the impacted canine and root resorption. Compared with conventional imaging, CBCT improves accuracy in localization and risk assessment [23], guiding prognosis and treatment selection.

Beyond tooth preservation, this conservative approach provided substantial functional and esthetic advantages compared with implant or prosthetic alternatives. Maintaining the natural incisor preserved alveolar bone height and gingival architecture, preventing infraocclusion and gingival recession commonly seen with implants in growing patients. Moreover, the retained natural tooth maintained proprioceptive function and a seamless emergence profile, resulting in superior esthetic and functional integration, in line with Holst et al. [14].

Although orthodontic treatment and esthetic restorations enhanced the result, the core achievement was the preservation of a central incisor that would traditionally have been considered hopeless. This case emphasizes the value of early CBCT‐based diagnosis, interdisciplinary planning, and minimally invasive endodontic surgery in achieving long‐term preservation despite severe resorption. Importantly, the 15‐year follow‐up underscores that conservative protocols can provide durable functional and esthetic outcomes, offering an alternative to extraction and prosthetic replacement in growing patients (Table 2).

**Table 2:** Clinical principles for managing high‐risk impacted canines.

Principle	Clinical rationale
Early CBCT diagnosis	Identifies proximity and resorption risk
Elimination of resorptive stimulus	Prevents progression
Conservative endodontic microsurgery	Preserves remaining root
Interdisciplinary planning	Optimizes functional outcome
Long‐term follow‐up	Confirms stability

## 4. Conclusion

This 15‐year case report shows that early CBCT‐guided diagnosis, elimination of the resorptive stimulus, and conservative interdisciplinary management can preserve severely compromised incisors in adolescents (Table 2). Even with retention of only one‐third of the root, periodontal support and function were maintained through root‐end sealing with MTA and careful occlusal management. Future prospective studies with larger cohorts are warranted to validate these findings and refine guidelines for managing similar high‐risk impactions.

This case reinforces that even teeth with advanced resorption should not be considered hopeless when early diagnosis and conservative interdisciplinary strategies are applied.

This case report was prepared in accordance with the CARE guidelines (Supporting Information File 1).

## Author Contributions

**Lara Maalouf:** conceptualization, methodology, investigation, data curation, project administration, writing – original draft, visualization, final approval. **Philip Farha:** writing – original draft, visualization, writing – review and editing. **Elie Amm:** investigation, data curation, formal analysis, visualization, writing – review and editing.

## Funding

This paper did not receive any specific grant from funding agencies in the public, commercial, or not‐for‐profit sectors.

## Ethics Statement

This case report was conducted in accordance with institutional ethical standards and the Declaration of Helsinki. Ethical approval was not required for a single retrospective case report. Written informed consent for treatment and publication of clinical data and images was obtained from the patient and her legal guardians.

## Conflicts of Interest

The authors declare no conflicts of interest.

## Supporting Information

Additional supporting information can be found online in the Supporting Information section.

## Supporting information


**Supporting Information** CARE Checklist Compliance Statement.

## Data Availability

Data sharing is not applicable to this article, as no datasets were generated or analyzed during the current study.

## References

[1] Kokich V. G. and Mathews D. P. , Surgical and Orthodontic Management of Impacted Teeth, Dental Clinics of North America. (1993) 37, no. 2, 181–204, 10.1016/S0011-8532(22)00276-2. 8477864

[2] Cruz R. M. , Orthodontic Traction of Impacted Canines: Concepts and Clinical Application, Dental Press Journal of Orthodontics. (2019) 24, no. 1, 74–87, 10.1590/2177-6709.24.1.074-087.bbo, 2-s2.0-85063951660. 30916252 PMC6434671

[3] Impellizzeri A. , Horodynski M. , and Serritell A. , Uncovering and Autonomous Eruption of Palatally Impacted Canines: A Case Report, Dentistry Journal. (2021) 9, no. 6, 2–8, 10.3390/dj9060066. PMC8229895 34207531

[4] Chang N. Y. , Park J. H. , and Lee M. Y. , et al.Orthodontic Treatment of Maxillary Incisors With Severe Root Resorption Caused by Bilateral Canine Impaction in a Class II Division 1 Patient, Journal of Clinical Pediatric Dentistry. (2016) 40, no. 2, 161–168, 10.17796/1053-4628-40.2.161, 2-s2.0-84973409261. 26950820

[5] Walker L. , Enciso R. , and Mah J. , Three-Dimensional Localization of Maxillary Canines With Cone-Beam Computed Tomography, American Journal of Orthodontics and Dentofacial Orthopedics. (2005) 128, no. 4, 418–423, 10.1016/j.ajodo.2004.04.033, 2-s2.0-26444548977. 16214621

[6] Kumar S. , Mehrotra P. , and Bhagchandani J. , et al.Localization of Impacted Canines, Journal of Clinical and Diagnostic Research. (2015) 9, ZE11–4, 10.7860/JCDR/2015/10529.5480, 2-s2.0-84924860130. 25738100 PMC4347191

[7] Ericson S. and Kurol J. , Early Treatment of Palatally Erupting Maxillary Canines by Extraction of the Primary Canines, The European Journal of Orthodontics. (1988) 10, no. 1, 283–295, 10.1093/ejo/10.1.283, 2-s2.0-0024146883. 3208843

[8] Cuminetti F. , Boutin F. , and Frapier L. , Predictive Factors for Resorption of Teeth Adjacent to Impacted Maxillary Canines, International Orthodontics. (2017) 15, no. 1, 54–68, 10.1016/j.ortho.2016.12.011, 2-s2.0-85011095638. 28159386

[9] Ericson S. and Kurol J. , Radiographic Examination of Ectopically Erupting Maxillary Canines, American Journal of Orthodontics and Dentofacial Orthopedics. (1987) 91, no. 6, 483–492, 10.1016/0889-5406(87)90005-9, 2-s2.0-0023356112. 3473928

[10] Liu D. G. , Zhang W. L. , Zhang Z. Y. , Wu Y. T. , and Ma X. C. , Localization of Impacted Maxillary Canines and Observation of Adjacent Incisor Resorption With Cone-Beam Computed Tomography, Oral Surgery, Oral Medicine, Oral Pathology, Oral Radiology, and Endodontology. (2008) 105, no. 1, 91–98, 10.1016/j.tripleo.2007.01.030, 2-s2.0-37249029881. 17507268

[11] Al Eryani F. A. , Al Akwa’a A. A. , Ishaq R. A. , and Al-Aizari N. A. , Association Between Maxillary Impacted Canine and Vertical and Sagittal Skeletal Patterns in a Group of Yemeni Adults, The Journal of Contemporary Dental Practice. (2025) 26, no. 2, 178–183, 10.5005/jp-journals-10024-3821. 40444513

[12] Kim G. Y. , Kim S. , Chang J. S. , and Pyo S. W. , Advancements in Methods of Classification and Measurement Used to Assess Tooth Mobility: A Narrative Review, Journal of Clinical Medicine. (2024) 13, no. 1, 10.3390/jcm13010142. PMC10779763 38202149

[13] Yan B. , Sun Z. , Fields H. , and Wang L. , Maxillary Canine Impaction Increases Root Resorption Risk of Adjacent Teeth: A Problem of Physical Proximity, L’Orthodontie Française. (2015) 86, no. 2, 169–179, 10.1051/orthodfr/2015014, 2-s2.0-84952315166. 26337094

[14] Holst S. , Hegenbarth E. A. , Schlegel K. A. , and Holst A. I. , Restoration of a Nonrestorable Central Incisor Using Forced Orthodontic Eruption, Immediate Implant Placement, and an All-Ceramic Restoration: A Clinical Report, The Journal of Prosthetic Dentistry. (2007) 98, no. 4, 251–255, 10.1016/S0022-3913(07)00266-1, 2-s2.0-35148818425. 17936123

[15] Utneja S. , Garg G. , Arora S. , and Talwar S. , Nonsurgical Endodontic Retreatment of Advanced Inflammatory External Root Resorption Using Mineral Trioxide Aggregate Obturation, Case Reports in Dentistry. (2012) 2012, 5, 10.1155/2012/624792, 624792. 23304567 PMC3530227

[16] Amer M. , Intracoronal Tooth Bleaching: A Review and Treatment Guidelines, Australian Dental Journal. (2023) 68, no. S1, 141–152, 10.1111/adj.13000. 37975331

[17] Chao Y. C. , Chen P. H. , and Su W. S. , et al.Effectiveness of Different Root-End Filling Materials in Modern Surgical Endodontic Treatment: A Systematic Review and Network Meta-Analysis, Journal of Dental Sciences. (2022) 17, no. 4, 1731–1743, 10.1016/j.jds.2022.05.013. 36299320 PMC9588816

[18] German D. S. , Chu S. J. , Furlong M. L. , and Patel A. , Simplifying Optimal Tooth-Size Calculations and Communications Between Practitioners, American Journal of Orthodontics and Dentofacial Orthopedics. (2016) 150, no. 6, 1051–1055, 10.1016/j.ajodo.2016.04.031, 2-s2.0-84997769488. 27894526

[19] Haak R. , Siegner J. , and Ziebolz D. , et al.OCT Evaluation of the Internal Adaptation of Ceramic Veneers Depending on Preparation Design and Ceramic Thickness, Dental Materials. (2021) 37, no. 3, 423–431, 10.1016/j.dental.2020.11.021. 33288325

[20] Chaushu S. , Kaczor-Urbanowicz K. , Zadurska M. , and Becker A. , Predisposing Factors for Severe Incisor Root Resorption Associated With Impacted Maxillary Canines, American Journal of Orthodontics and Dentofacial Orthopedics. (2015) 147, no. 1, 52–60, 10.1016/j.ajodo.2014.09.012, 2-s2.0-84919667513. 25533072

[21] Lai C. S. , Bornstein M. M. , Mock L. , Heuberger B. M. , Dietrich T. , and Katsaros C. , Impacted Maxillary Canines and Root Resorptions of Neighbouring Teeth: A Radiographic Analysis Using Cone-Beam Computed Tomography, The European Journal of Orthodontics. (2013) 35, no. 4, 529–538, 10.1093/ejo/cjs037, 2-s2.0-84880999468. 22828076

[22] Pitt S. , Hamdan A. M. , and Rock W. P. , A Treatment Difficulty Index for Unerupted Maxillary Canines, European Journal of Orthodontics. (2006) 28, no. 2, 141–144, 10.1093/ejo/cji068, 2-s2.0-33645463673. 16043468

[23] Farha P. , Nguyen M. , Karanth D. , Dolce C. , and Arqub S. A. , Orthodontic Localization of Impacted Canines: Review of the Cutting-Edge Evidence in Diagnosis and Treatment Planning Based on 3D CBCT Images, Turkish Journal of Orthodontics. (2023) 36, no. 4, 261–269, 10.4274/TurkJOrthod.2023.2022.131. 38164014 PMC10763599

