# Supplementary material for: 15‐Year Follow‐Up of Multidisciplinary Management of Severe External Root Resorption Caused by Biocortically Impacted Maxillary Canine: A Case Report
Source: Case Rep Dent. 2026 Feb 26;2026:5169817. doi: 10.1155/crid/5169817 (PMC12946475; doi:10.1155/crid/5169817)
Supplement: Supplementary file 1 — Supporting Information CARE Checklist Compliance Statement. [file CRID-2026-5169817-s001.docx]

| 1 | Title identifies the article as a case report | Yes | Title Page |
| --- | --- | --- | --- |
| 2 | Key words | Yes | Abstract-Keywords |
| 3 | Abstract | Yes | Abstract |
| 4 | Introduction- Background and Rationale | Yes | Introduction |
| 5 | Patient Information (demographics, main concerns, medical history) | Yes | Case Report |
| 6 | Clinical Findings | Yes | Case Report |
| 7 | Timeline | Yes | Table 1 |
| 8 | Diagnostic Assessment (diagnostic methods, challenges, reasoning) | Yes | Case Report |
| 9 | Therapeutic Intervention (types, administration, rationale) | Yes | Treatment Management |
| 10 | Follow-up and outcomes (clinical and radiographic results) | Yes | Outcomes |
| 11 | Patient Perspective | Yes | Outcomes |
| 12 | Informed Consent | Yes | Patient consent Statement |
| 13 | Ethical Approval | Yes | Ethics Statement |
| 14 | Discussion (strengths, limitations, literature comparison) | Yes | Discussion |
| 15 | Funding | Yes | Funding statement |
| 16 | Conflict of Interest | Yes | Conflict of interest |

CARE Checklist Compliance Statement
